# MRI in the Diagnosis of Transient Global Amnesia: A Case Series and Review of Current Evidence

**DOI:** 10.7759/cureus.83642

**Published:** 2025-05-07

**Authors:** Saeedur Rahman, Jafor Sadeque, Garryck Tan, Jesmin Rahman

**Affiliations:** 1 Department of Stroke Medicine, Dartford and Gravesham NHS Trust, Dartford, GBR; 2 Department of Radiology, Dartford and Gravesham NHS Trust, Dartford, GBR; 3 Department of Acute Medicine, Colchester General Hospital, Colchester, GBR

**Keywords:** acute confusional state, amnesia, punctate hippocampus lesion, stroke, transient global amnesia

## Abstract

Transient global amnesia (TGA) is a common acute amnestic syndrome characterised by sudden-onset predominant anterograde amnesia lasting up to 24 hours. Patients with TGA frequently ask repetitive questions reflecting disorientation and may have some degree of inability to recall general or personal information (retrograde amnesia) while the episode lasts. During the episode of TGA, other cognitive functions are normal. Episodes are self-limited and, by definition, resolve within 24 hours, with recovery of memory function symptoms, except for what happened during the episode. Although diagnosis is primarily clinical, neuroimaging plays a crucial role in excluding alternative causes. Recent evidence suggests that characteristic findings on diffusion-weighted imaging (DWI) may support the diagnosis of TGA. Here, we present three cases of TGA evaluated at Darent Valley Hospital, Kent, UK, with acute onset of confusion and anterograde memory loss. Case 1 was a 53-year-old man who presented to the emergency department with a new onset of confusion, which completely resolved within three hours. Subsequent MRI of the brain showed a punctate area of restricted diffusion involving both hippocampi, which later fully resolved without any residual damage on a follow-up MRI after one week. Case 2 was a 63-year-old woman who became acutely confused while working as a caterer organising a large event. The episode lasted for approximately six to seven hours. MRI of the brain showed a punctate area of restricted diffusion involving the right hippocampus, which fully resolved on an interval MRI scan performed two weeks later. Case 3 was a 59-year-old man who was brought to the hospital due to the sudden onset of confusion noticed by his wife, which lasted for 12 hours. An initial MRI of the brain, conducted four hours after symptom onset, showed no definitive abnormalities. A repeat scan 24 hours after symptom onset revealed an interval appearance of restricted diffusion involving the tail and head of the right and left hippocampi, respectively. All three cases were diagnosed as TGA based on their clinical presentation and MRI findings. The prevalence of typical MRI findings related to TGA varies widely and has been reported to be as high as 85% in some studies. However, these findings may be underdetected without a high index of clinical suspicion from the radiologist. MRI of the brain, in conjunction with clinical history, can improve diagnostic confidence in cases of TGA.

## Introduction

Amnesia is now understood as a selective memory impairment rather than total memory loss, with different types of memory relying on distinct neuroanatomical structures. Tulving’s classification divides long-term memory into hierarchical systems, including episodic-autobiographical memory (EAM) for personal experiences and semantic memory for factual knowledge [1]. Anterograde amnesia prevents new memory formation, while retrograde amnesia affects recall, often affecting recent memories more (Ribot’s law).

Amnestic disorders are classified in the ICD-10 (International Classification of Diseases, 10th Revision) and DSM-IV-TR (Diagnostic and Statistical Manual of Mental Disorders, fourth edition, text revision) based on medical, substance-induced, or psychological causes. While terms such as dissociative, psychogenic, and functional amnesia are often used interchangeably, they have distinct differences. EAM is typically the most affected.

Transient global amnesia (TGA) is one of the most frequently encountered acute amnestic syndromes. TGA is a distinct clinical syndrome characterised by the sudden onset of anterograde amnesia, lasting up to 24 hours, while other cognitive functions remain intact [2]. Patients often exhibit repetitive questioning and temporary retrograde amnesia, but memory typically recovers except for the events during the episode. The exact cause is unknown, though proposed mechanisms include vascular, migraine, epileptic, and psychogenic factors [3,4]. While some patients may experience recurrent episodes, TGA has a benign prognosis, and management focuses on diagnosis, ruling out other causes, and patient reassurance. The diagnosis of TGA is mostly clinical and involves ruling out alternative diagnoses, both organic and functional. The main differential diagnoses of TGA are transient ischaemic attack (TIA), transient epileptic amnesia (TEA), and psychogenic or dissociative amnesia [5,6]. 

The role of neuroimaging in these cases has traditionally been to rule out alternative diagnosis. However, MRI brain can show distinctive changes in brain parenchyma that can be utilised (in addition to clinical features) to help make a definitive diagnosis of TGA. The prototypical MRI findings in TGA have different nomenclature, with hippocampal restricted diffusion (HRD) being the most popular. These are small subcentimetre areas of restricted diffusion (visible on specific MRI sequences: diffusion-weighted imaging (DWI) and apparent diffusion coefficient (ADC)) involving various parts of the hippocampal formation. Their detection is highly dependent on the timing of the imaging [7]. A typical history, along with classical radiological findings on MRI, can increase diagnostic confidence and help clinicians differentiate TGA from other pathologies. We report three cases seen at Darent Valley Hospital, UK, highlighting the imaging findings and further discussing the use of MR imaging in such cases.

## Case presentation

Case 1

A 52-year-old man was brought to the emergency department (ED) by his wife after a sudden onset of confusion and memory loss, which began approximately two hours earlier. He had no significant past medical history. According to his wife, he had been completely well earlier in the day. However, upon her return home, she found him disoriented, with a vacant expression and no awareness of the time. Strikingly, he had forgotten to collect his children from school, an uncharacteristic lapse. His wife also mentioned that although he was physically well, he had been under immense work-related stress, leading her to believe that he might be having a mental breakdown.

During this episode, he repeatedly asked the same questions about dates, months, and the whereabouts of his children, despite being given answers multiple times. Upon presentation to the ED, he remained disoriented, scoring 5 out of 10 on the Abbreviated Mental Test-10 (AMT-10) and 14/15 on the Glasgow Coma Scale (GCS) due to confusion. He was unable to recall his date of birth and his profession. He was able to engage with the medical staff, and his neurological examination was otherwise unremarkable.

Within an hour of arrival, the patient regained full orientation; however, he exhibited complete amnesia for the events of the preceding few hours. He denied any associated symptoms such as dizziness, lightheadedness, chest pain, palpitations, dyspnoea, or headache. His wife reported no witnessed seizure-like activity, including jerking movements or incontinence, and there was no history of similar episodes in the past.

In view of his acute amnestic presentation, a broad range of differentials was considered, including metabolic disorder and TIA. Routine blood investigations, including complete blood count, metabolic panel, and inflammatory marker such as CRP, were all within normal limits. A non-contrast CT head scan was performed as part of the local ED protocol as an initial imaging for suspected TIA. The CT head did not reveal any abnormal findings.

Given the acuteness of the amnestic symptoms, the patient was referred to the stroke team to exclude a cerebrovascular event. His National Institutes of Health Stroke Scale (NIHSS) score was 0, and at that point, he had returned to his cognitive baseline with complete resolution of symptoms. TIA was considered a less likely diagnosis compared to a possible diagnosis of TGA.

**Figure 1:**
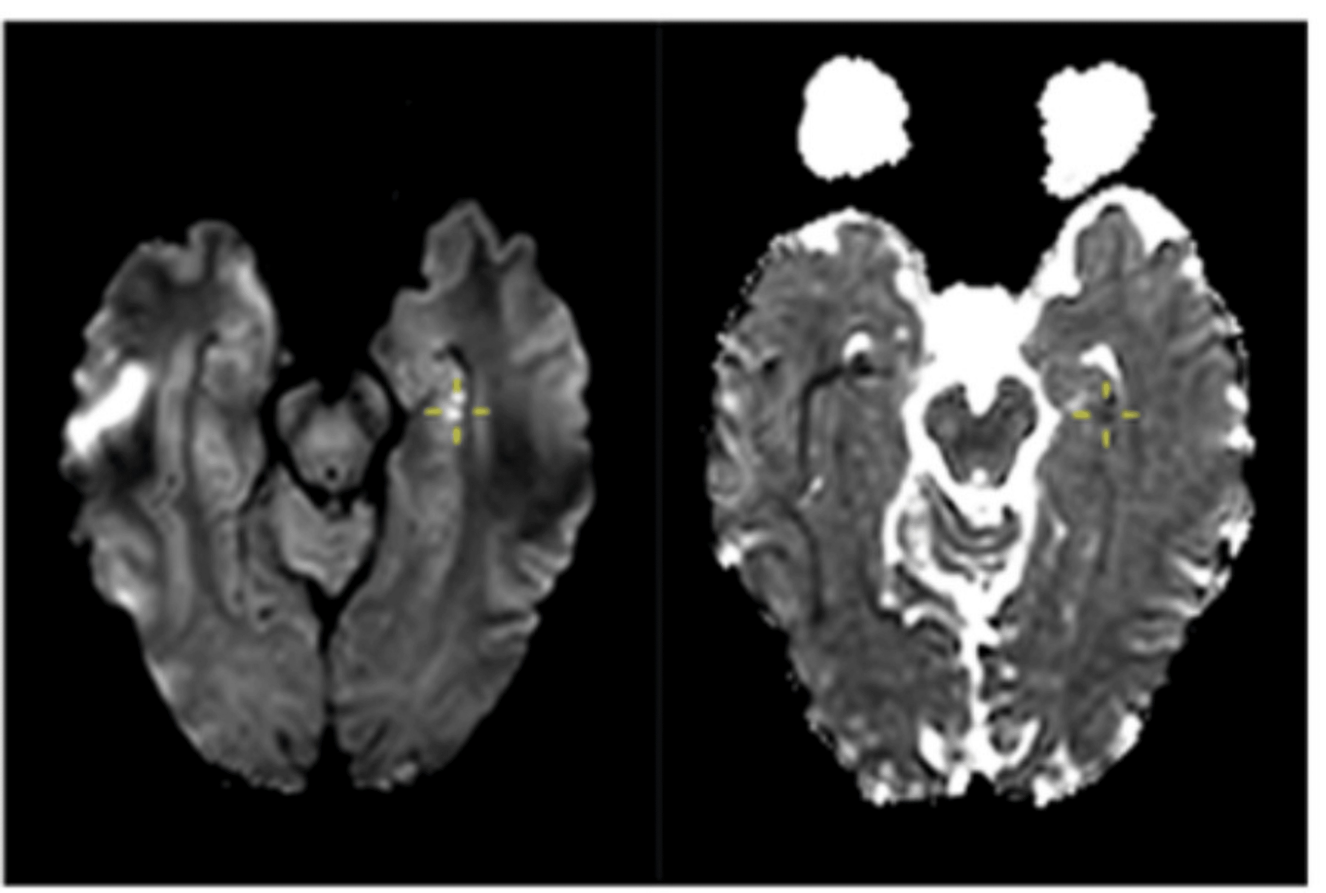
Case 1: Punctate area of restricted diffusion affecting the left hippocampal head (cross-marked).

**Figure 2:**
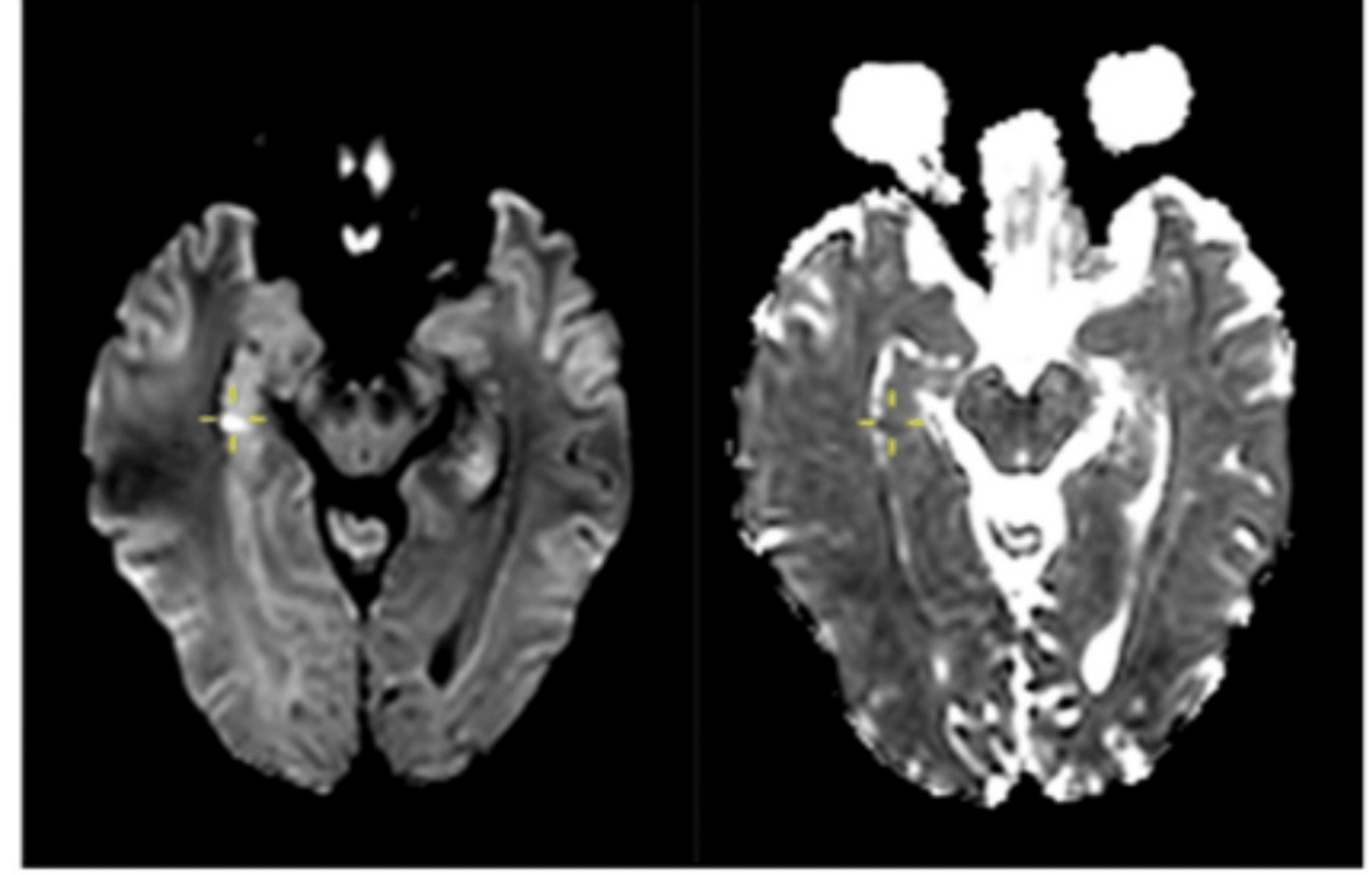
Case 1: Punctate area of restricted diffusion affecting the right hippocampal tail of the same patient (cross-marked).

An MRI brain was performed to further evaluate the unexplained transient neurological disturbance. The MRI revealed punctate hyperintensities on DWI with corresponding hyperintensities on ADC mapping, involving both hippocampi (Figures 1, 2).

As areas of restricted diffusion can signify ischaemic changes, patient was discharged on antiplatelet agent, and a follow-up outpatient MRI was arranged after one week. His repeat MRI showed complete resolution of hyperintensities in both hippocampi. In case of an ischaemic lesion, one would expect the DWI lesion to be less hyperintense with resolving ADC hypointensity. It also showed no residual parenchymal changes on 3D FLAIR bilaterally, which would otherwise be present in cases of ischaemic lesions. This allowed for differentiation from an acute vascular event. TGA was the final diagnosis. The patient was reassured, and antiplatelet treatment was discontinued.

Case 2

A 63-year-old woman with a history of hypothyroidism was brought to the ED by her daughter following an acute episode of confusion. According to her daughter, the patient had experienced headache the previous day while preparing for a large catering event. She was quite tired and exhausted after working throughout the day. Shortly thereafter, she became forgetful and began exhibiting repetitive questioning. She appeared disoriented and was unable to locate personal items, such as her shoes and handbag.

By the time she was assessed in the ED, her symptoms had completely resolved. She scored 10/10 on the AMTS and 15/15 on the GCS. A thorough neurological examination was normal. However, the patient was amnestic for the events that had occurred during the episode, which lasted approximately six to seven hours in total. A non-contrast CT brain was performed and showed no acute abnormalities. Routine blood investigations, including thyroid function tests, were within normal limits.

**Figure 3:**
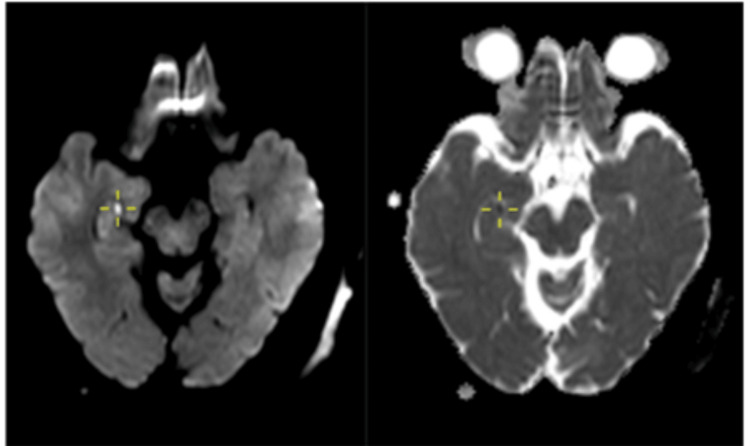
Case 2: Image on the left shows a punctate DWI lesion involving the right hippocampus with a corresponding area of low ADC on the right image (cross-marked). DWI: diffusion-weighted imaging; ADC: apparent diffusion coefficient.

The stroke team was consulted to evaluate a possible vascular event. The patient had an NIHSS score of 0. A subsequent MRI brain revealed a small punctate hypointensity on DWI with a corresponding low signal on the ADC map, localised to the right hippocampus (Figure 3).

The patient was started on antiplatelet therapy. Over the next 24 hours, she remained clinically stable and asymptomatic. As her presentation was more in favour of TGA, she was discharged home the following day with a follow-up MRI scheduled in two weeks.

**Figure 4:**
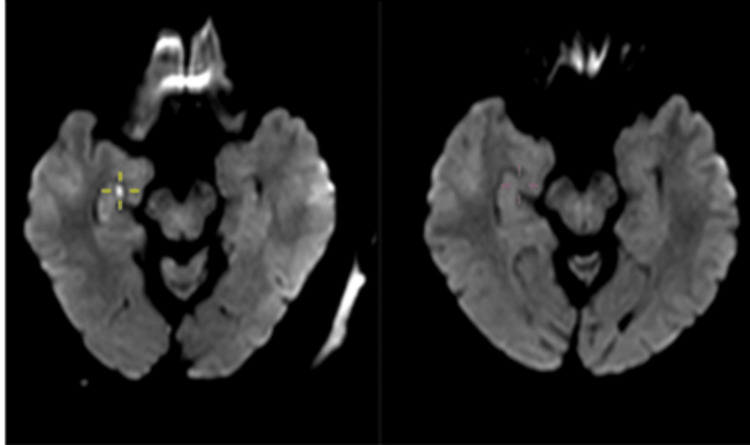
Case 2: Image on the left side shows a small area of restricted diffusion (cross-marked) in the right hippocampal head. Image on the right side shows complete resolution of the area of restricted diffusion on a follow-up MRI.

The outpatient MRI performed two weeks later showed resolution of the previously noted DWI lesion, with no abnormalities on 3D FLAIR sequences (Figure 4). This helped to differentiate it from an ischaemic event. Based on the clinical presentation and imaging findings, a diagnosis of TGA was established. Antiplatelet therapy was subsequently discontinued.

Case 3

A 59-year-old man with no known prior medical history was brought to the ED by ambulance following a sudden onset of confusion, first noticed by his wife. He appeared disoriented and repeatedly asked the same question, seemingly unaware of his surroundings. His wife reported no associated symptoms such as slurred speech, limb weakness, or convulsive movements.

On arrival to the ED, he remained confused, with an AMTS of 6 out of 10 and a GCS of 14/15 due to confusion. He was able to follow instructions, and his neurological examination revealed no focal deficits.

All routine blood tests, including blood sugar and renal function tests, were normal. He underwent a non-contrast CT head as per local ED protocol, which revealed a normal appearance of the brain parenchyma. His NIHSS was 2 due to an inability to answer questions such as “month of year” and “age” correctly. An initial MRI brain was performed approximately four hours after symptom onset, which did not show any abnormalities. The patient was admitted for observation and further evaluation.

**Figure 5:**
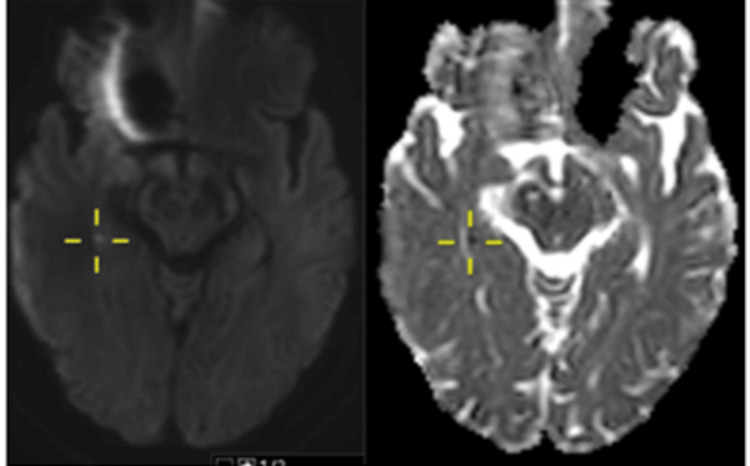
Case 3: Punctate area of restricted diffusion involving the right hippocampal tail (cross-marked).

**Figure 6:**
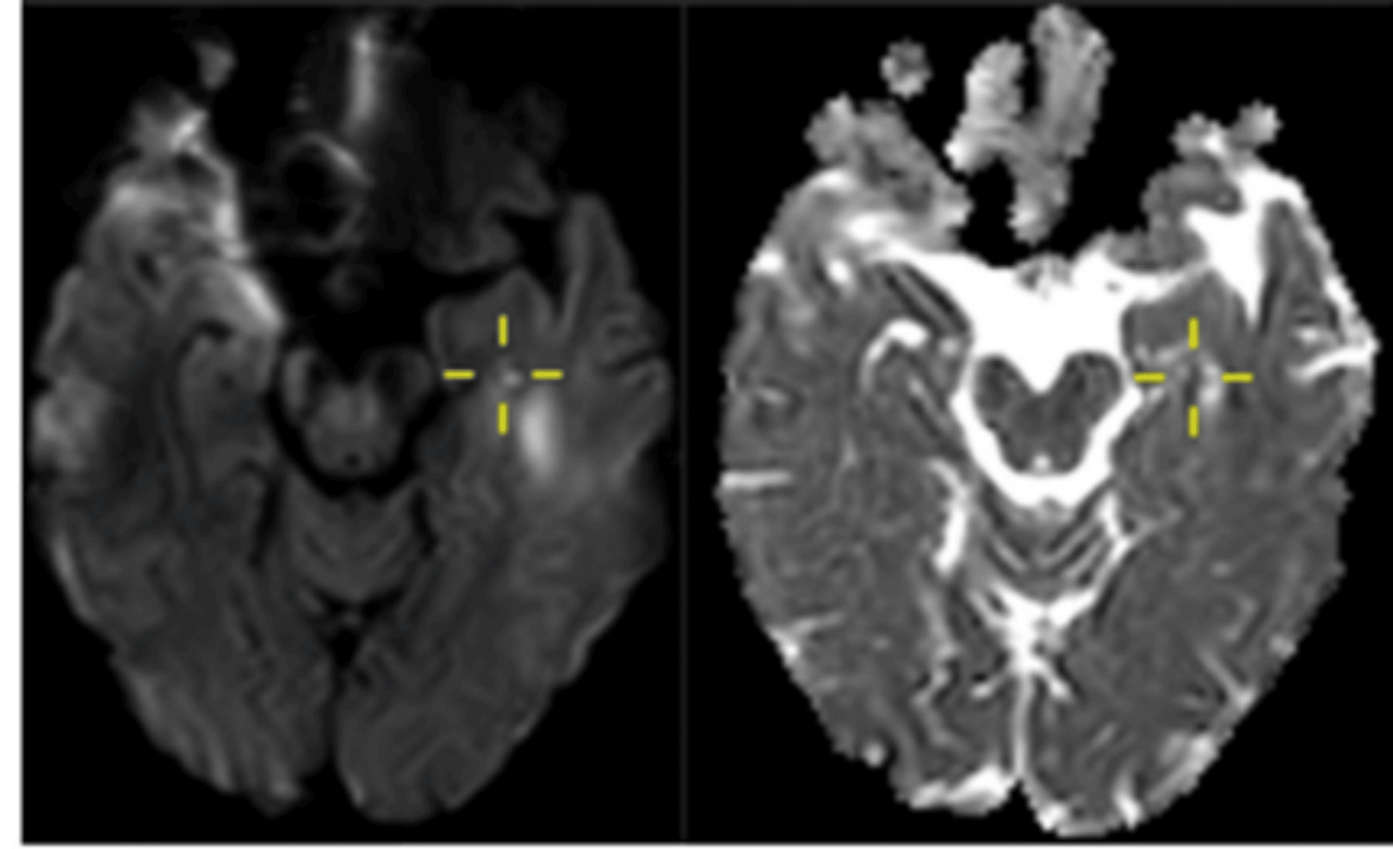
Case 3: Punctate area of restricted diffusion involving the left hippocampal head (cross-marked).

Over the next 12 hours, his symptoms gradually resolved. Given the clinical suspicion of TGA, a repeat MRI brain was scheduled and performed 24 hours after symptom onset. This scan revealed punctate areas of restricted diffusion involving the tail of the right hippocampus and the head of the left hippocampus, without any associated signal changes on FLAIR sequences (Figures 5, 6). Based on the characteristic clinical presentation and the “delayed” appearance of restricted diffusion in the bilateral hippocampi (this is in contrast with an acute ischaemic lesion, which will be apparent immediately), a diagnosis of TGA was confirmed. The patient was discharged with reassurance, and no further intervention was considered necessary.

## Discussion

Epidemiology, incidence, and risk factors of TGA

TGA is one of the most well-known acute amnestic disorders. It is a dramatic but benign disorder with no long-term sequelae. TGA has an annual incidence of 5.2 to 10 per 100,000 in the general population, increasing to 23.5 to 32 per 100,000 in those over 50 years [8]. It primarily affects adults aged 50 to 80, with a mean onset of 60 to 65 years, and occurs equally in both sexes, though some studies suggest a slight female predominance.

The link between vascular risk factors and TGA remains controversial, with mixed findings on hypertension, diabetes, and hypercholesterolemia. Migraine has been strongly associated with TGA in some studies, especially in recurrent cases. Other possible risk factors include familial occurrence (linked to familial migraine) and obstructive sleep apnoea, reported in 45% of cases in a small study [3,5,9].

Clinical criteria for diagnosis

The diagnosis of TGA remains mostly clinical, with some following the diagnostic criteria proposed by Hodges and Warlow [10]. These are entirely based on clinical history and examination findings. Table 1 shows the Hodges and Warlow’s clinical criteria for the diagnosis of TGA and how it can be applied to our three cases.

**Table 1:** Hodges and Warlow's criteria for the diagnosis of TGA and comparison of three cases. TGA: transient global amnesia.

Clinical criteria	Case 1	Case 2	Case 3
Attacks must be witnessed from a capable observer	Yes	Yes	Yes
There must be anterograde amnesia during the attack	Yes	Yes	Yes
There must be no clouding of consciousness or loss of identity	Yes	Yes	Yes
	Not orientated to time and place		Not orientated to time and place
Cognitive impairment must be limited to amnesia (no apraxia or aphasia)	Yes	Yes	Yes
There must be no focal neurological signs and symptoms before or after the attack	Yes	Yes	Yes
There must be no epileptic features	Yes	Yes	Yes
Attacks must be resolved within 24 hours	Yes	Yes	Yes
There must be no recent head injury or active epilepsy	Yes	Yes	Yes

TGA attacks can be triggered by various factors such as physical and emotional stress, headache/migraine attacks, alcohol binge, hot bath, etc. The duration of an attack may vary widely. However, it invariably resolves within 24 hours. Patients do not show any focal neurological deficits, and there are no long-term sequelae. TGA is a monophasic disorder. Symptoms lasting longer than 24 hours, presence of focal neurological signs, and recurrent episodes are all suggestive of a possible alternative diagnosis [6,11]. Table 2 summarises the variation in their presentations for the three cases.

**Table 2:** Comparison of clinical presentations of the three patients with TGA. TGA: transient global amnesia.

Clinical presentation	Case 1	Case 2	Case 3
Age	52	63	59
Sex	Male	Female	Male
Trigger factor	Emotional stress	Physical stress	None
Duration of symptoms	3 hours	6 to 7 hours	12 hours
Presence of anterograde amnesia	Present	Present	Present
Presence of retrograde amnesia	Present to some degree	Absent	Absent
Other neurological symptoms	Absent	Absent	Absent
Vascular risk factors	None	None	None
Other neurological disorders, including epilepsy	None	None	None
Previous similar episodes	None	None	None

Differential diagnoses of TGA

There are a number of differential diagnoses of acute amnestic attack. TIA, TEA, and psychogenic or dissociative amnesia are three other major differential diagnoses other than TGA. A careful history and physical examination are the key (Table 3).

**Table 3:** Features of various differential diagnoses of TGA. TGA: transient global amnesia; TIA: transient Ischaemic attack; TEA: transient epileptic amnesia.

Features	TIA	TEA	Psychogenic or dissociative amnesia	TGA
Onset	Sudden	Sudden, often upon awakening	Often sudden, triggered by emotional stress	Sudden, often triggered by stress, exertion, or pain
Duration	Normal or non-specific	Often abnormal (interictal discharges in mesial temporal lobes)	Normal	Normal
Type of memory affected	Variable; often anterograde with other deficits	Mainly retrograde amnesia during ictus	Retrograde autobiographical memory loss	Primarily anterograde amnesia with patchy retrograde loss
Repetitive questioning during attack	Rare	Uncommon	Rare	Very common
Other neurological symptoms	Often present (e.g., weakness, visual field deficits)	May have olfactory hallucinations, automatisms	No neurological signs	None, apart from memory impairment
Recurrence	Possible (depends on vascular risk)	Common, multiple episodes	Possible	Rare
Risk factors	Vascular (hypertension, AF, atherosclerosis)	Epilepsy history, seizures	Psychological stress, trauma	None clear; possibly migraine, stress-related
Triggers	No definitive triggers	Similar to epilepsy	Often triggered by psychological and emotional stress	Often triggered by emotional, physical stress, alcohol binge, hot bath, etc.

TEA can mimic TGA closely. The Zeman Criteria for diagnosing TEA were proposed by Professor Adam Zeman and colleagues to help differentiate TEA from other transient amnesic syndromes like TGA [6]. The Zeman Criteria for diagnosing TEA include recurrent, transient amnesia episodes lasting 30 to 60 minutes, often upon waking. During episodes, patients retain their identity and cognitive abilities. Diagnosis is supported by evidence of epilepsy, such as EEG abnormalities, response to antiepileptic drugs, or epilepsy-related symptoms.

TIA or ischaemic stroke can present with amnestic symptoms; however, a careful history and physical examination reveal other associated focal neurological symptoms and signs, such as temporal lobe dysfunction like visual field defects. The presence of cardiovascular risk factors is an important clue in dealing with such cases. Neuroimaging, such as MRI, can often reveal areas of restricted diffusion during acute and subacute phases with corresponding FLAIR hyperintense lesion in areas such as mesial temporal lobe, thalamus, caudate head and cingulate gyrus [3].

In psychogenic or dissociative amnesia, retrograde autobiographical memory is profoundly affected to a degree that one may even forget his/her name and often find themselves in a fugue state (retrograde memory loss and wandering into unknown/unfamiliar place) [12].

Use of MRI in diagnosis of TGA

We believe that MRI lesions distinctive of TGA are an underutilised and less known diagnostic marker among clinicians. HDR is the hallmark MRI finding of TGA. Diffusion restriction in the hippocampus can be seen in as many as 84% of patients with TGA in different populations depending on the timing of the imaging, strength of the MRI machine, appropriate protocol, and high pre-test probability among the reporting radiologist [13]. However, various retrospective studies have shown that in clinical practice, generally 30 to 35% cases of TGA shows typical MRI lesions [14].

Imaging technique, timing, and detection rate of HDR in TGA

HDR detection is highly dependent on the timing and strength of the MRI machine and protocol used. A large multicentre cohort study has shown that 1.5 T or 3T MRI scanners acquiring standard DWI imaging with 3 mm slices have the best detection rate [13]. One of the major differences of HDR, which is related to TGA and not ischaemic, is the delayed appearance of the lesions. Typically, an ischaemic lesion will give rise to a positive DWI and ADC appearance immediately. On the contrary, in the case of TGA, diffusion restriction mostly appears after 12 hours of symptom onset and peaks maximum between 24 and 84 hours [13]. Another study has shown the peak to be between 12 and 24 hours [15].

Location, distribution, and nature of HDR in TGA

Anatomically, the hippocampus is divided into head, body, and tail, whereas histologically it is divided into four zones, CA1 to CA4 (Cornu ammonis). This is based on sensitivity to hypoxia where CA1 is most vulnerable and CA3 is most resistant [16]. The distribution of these histological zones varies in relation to different anatomical portions of the structure. The hippocampal head predominantly consists of CA3, while body and tail predominantly consist of the CA1 zone. This has implications for the distribution of HDR in TGA.

**Figure 7:**
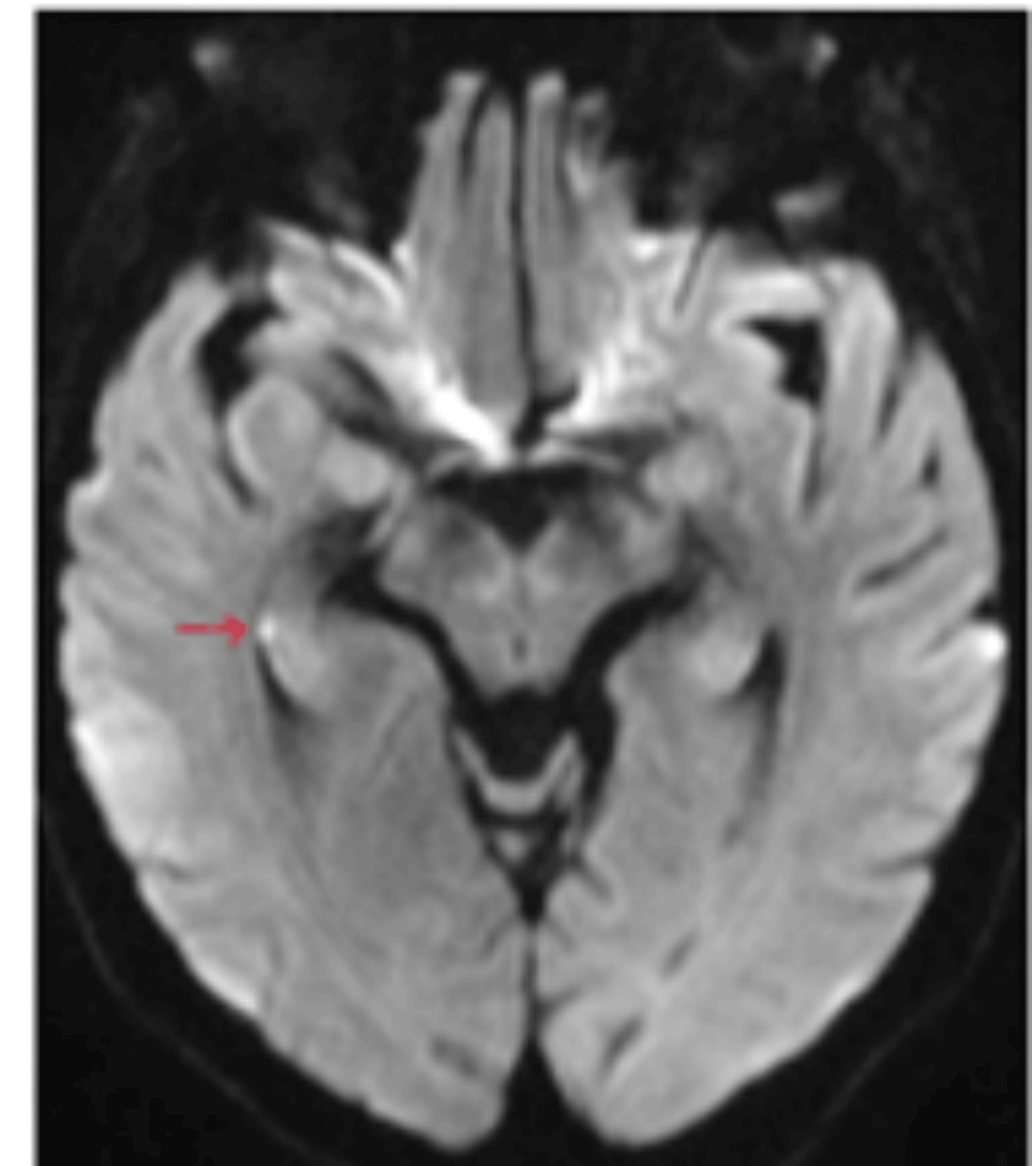
Area of restricted diffusion in the right hippocampal body (red arrow). Case courtesy of Alan Coulthard, Radiopaedia.org, rID 37792 [17]. Copyright/License: This image is available under a Creative Commons Non-Commercial Attribution, Sharealike license (CC-NC-BY-SA 3.0).

**Figure 8:**
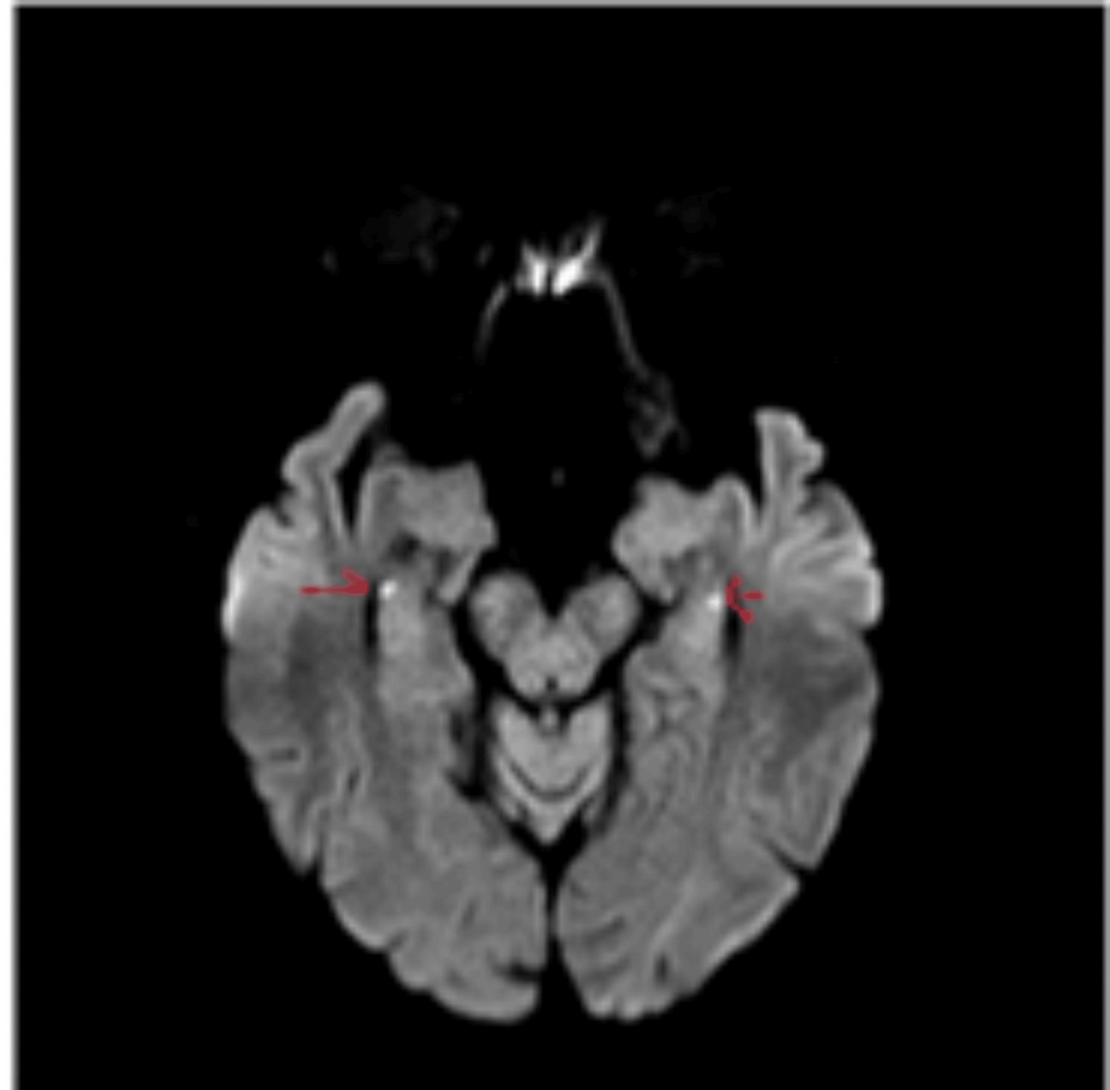
Area of restricted diffusion involving bilateral hippocampal head (red arrows). Case courtesy of Salvatore Belluardo, Radiopaedia.org, rID 75185 [18]. Copyright/License: This image is available under a Creative Commons Non-Commercial Attribution, Sharealike license (CC-NC-BY-SA 3.0).

**Figure 9:**
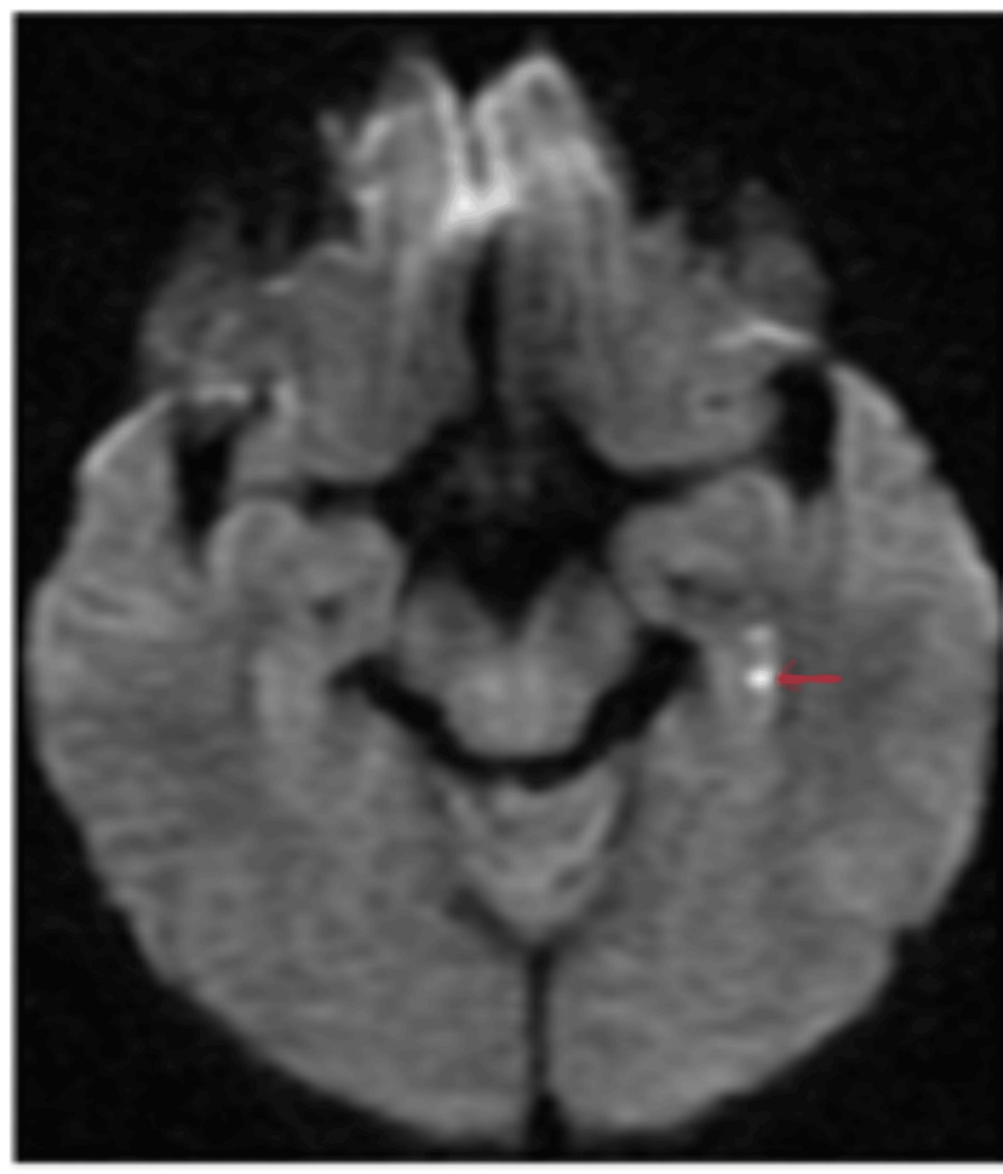
Area of restricted diffusion in left hippocampal tail (red arrow). Case courtesy of Rodrigo Dias Duarte, Radiopaedia.org, rID 60129 [19]. Copyright/License: This image is available under a Creative Commons Non-Commercial Attribution, Sharealike license (CC-NC-BY-SA 3.0).

HDR is mostly unilateral (80%) [13,14]. However, it can be seen bilaterally as well. Lesions can be single or multiple, with majority of patients showing a single lesion. Most lesions are located in the hippocampal body, followed by tail and head, which corresponds to the histological makeup (CA1 to CA4) of these parts as described above (Figures 7-9).

Extrahippocampal lesions are rare [7]. Studies have not identified any correlation between the locations of the HDR and the clinical presentation. It is a well-known fact that the majority of the TGA are triggered by emotional or physical stress, and one study showed a weak correlation between lesions in the hippocampal tail and emotional stress as a trigger factor [13].

These lesions are typically 1 to 3 mm in diameter and hyperintense on DWI with corresponding reduction in ADC. There are often corresponding T2 and FLAIR signal changes during the initial imaging. As a rule, all imaging abnormalities resolve completely beyond one week of symptom onset in most cases. Table 4 summarizes all the relevant information.

**Table 4:** Summary of MRI imaging protocol and findings in TGA/characteristics of HDR. HDR: hippocampal diffusion restriction; TGA: transient global amnesia.

Imaging modality	MRI brain
Ideal MRI scanner	1.5 to 3 Tesla
MRI protocol	MRI protocol including DWI, ADC, FLAIR. 3 mm slices
Timing of imaging	Mostly positive after 12 hours of symptom onset and peaks between 24 and 84 hours
Location of HDR	Mostly in hippocampal body followed by tail and head
Shape and size	Punctate 1 to 3 mm lesions
Typical MRI appearance	Hyperintense of DWI with matched reduction or hypointensity on ADC. Negative FLAIR
Unilateral vs Bilateral	Mostly unilateral in 80% MRI positive cases
Extrahippocampal lesion	Rare
Long-term radiological sequelae	Hallmark of HDR in TGA is the complete resolution of diffusion restriction with no FLAIR abnormality

Other hippocampal DWI lesions and underlying pathology

There are a number of other disease conditions, which can give rise to restricted diffusion in the hippocampus, most notably ischaemic stroke, limbic encephalitis, seizures, and transient epileptic amnesia. Neuroinflammatory disorders like Behcet’s disease and other non-neurological disorders like heat stroke can also cause similar appearance [20]. As a rule, the hippocampal lesions in these cases are more diffuse compared to the punctate TGA lesions [10].

## Conclusions

TGA is one of the common acute amnestic syndromes. Although a careful clinical history and examination are key to diagnosis, MRI can be a useful adjunct not only for ruling out other differentials but also for confirming the diagnosis of TGA. Due to the subtle nature of the findings, the radiologist needs to be offered relevant information. Many times, these lesions are identified on subsequent reviews, and there is a significant false negative result in the initial reporting. It is important to remember that relying solely on imaging finding may result in a falsely low diagnosis and the clinical history and physical examination remain more important. Ongoing research is needed to further improve our understanding in this area.
